# Materials Characterization of Feraheme/Ferumoxytol and Preliminary Evaluation of Its Potential for Magnetic Fluid Hyperthermia

**DOI:** 10.3390/ijms140917501

**Published:** 2013-08-26

**Authors:** John P. Bullivant, Shan Zhao, Brad J. Willenberg, Bettina Kozissnik, Christopher D. Batich, Jon Dobson

**Affiliations:** 1 Department of Materials Science & Engineering, University of Florida, Gainesville, FL 32611-6400, USA; E-Mails: jpbulli@ufl.edu (J.P.B.); shanzhao401@gmail.com (S.Z.); yoda@ufl.edu (B.J.W.); cbati@mse.ufl.edu (C.D.B.); 2 J. Crayton Pruitt Family Department of Biomedical Engineering, University of Florida, Gainesville, FL 32611-6131, USA; E-Mail: b.kozissnik@ufl.edu; 3 Institute for Cell Engineering and Regenerative Medicine, University of Florida, Gainesville, FL 32611-6131, USA

**Keywords:** Feraheme, magnetic fluid hyperthermia, magnetic nanoparticles, MRI contrast

## Abstract

Feraheme, is a recently FDA-cleared superparamagnetic iron oxide nanoparticle (SPION)-based MRI contrast agent that is also employed in the treatment of iron deficiency anemia. Feraheme nanoparticles have a hydrodynamic diameter of 30 nm and consist of iron oxide crystallites complexed with a low molecular weight, semi-synthetic carbohydrate. These features are attractive for other potential biomedical applications such as magnetic fluid hyperthermia (MFH), since the carboxylated polymer coating affords functionalization of the particle surface and the size allows for accumulation in highly vascularized tumors via the enhanced permeability and retention effect. This work presents morphological and magnetic characterization of Feraheme by transmission electron microscopy (TEM), Energy dispersive X-ray spectroscopy (EDX), and superconducting quantum interference device (SQUID) magnetometry. Additionally, the results of an initial evaluation of the suitability of Feraheme for MFH applications are described, and the data indicate the particles possess promising properties for this application.

## 1. Introduction

Superparamagnetic iron oxide nanoparticle (SPION) contrast agents for MRI generally consist of a biocompatible polymer coating or matrix with superparamagnetic (ferri- or ferromagnetic) iron oxide cores [1–3]. Superparamagnetic particles are nanoscale particles in which quantum effects dominate over bulk effects. For iron oxides, this generally translates to diameters of less than 30 nm. They exhibit strong magnetization in the presence of an applied field; however, upon removal of the field, they retain no permanent magnetization, making them ideal for *in vivo* applications where magnetic dipole-induced aggregation of particles within the vasculature would have the potential to cause embolization.

Inside the scanner, the SPIONs cause local perturbations of the magnetic field around the particles, resulting in detectable variations of the relaxation times of nearby proton spins after a probing RF pulse has been applied. Specifically, these local distortions increase proton dephasing, providing contrast enhancement in T_2_-weighted (spin-spin or transverse) MR images. In fact, the resulting T_2_-weighted images show signal intensity decreases, known as negative contrast, in regions where the SPIONs have aggregated [4].

Although many of these particles eventually end up naturally in the liver, the leaky vasculature present in solid tumors also promotes extravasation and accumulation—an effect known as “enhanced permeability and retention (EPR)—thereby providing contrast highlighting of the tumor against the surrounding tissue. The contrast effects are amplified when reactive cells associated with tumors also take up the particles (e.g., [5,6]). Implanted or injected cells that have been doped with SPIONs also can be tracked via T_2_-weighted MR imaging for applications in regenerative medicine and cell targeting (For review see [7]).

Though FDA-cleared, the main commercially available agents, such as Feridex/Comidex, have been withdrawn from the market [8]. Recently, a new SPION-based MRI contrast agent, Feraheme (also known as Ferumoxytol—AMAG Pharmaceuticals), has received a significant amount of attention due to its FDA-cleared status for use in humans, and there is keen interest to understand its magnetic properties. Feraheme is a SPION coated with a low molecular weight semi-synthetic carbohydrate. It is particularly indicated for the treatment of iron deficiency anemia in adult patients with chronic kidney disease [9]. Feraheme has a hydrodynamic diameter of *ca*. 30 nm and, most importantly for other potential biomedical applications, the carboxylated polymer coating allows for the bio-functionalization of the particle’s surface [10].

To date, the molecular weight and iron release profile of feraheme have been investigated [11], as have some of the basic magnetic properties [10,12], and these studies have focused on bio-functionalization and MRI contrast. A thorough evaluation of the magnetic properties of Feraheme and consideration of its potential use in other biomedical applications, such as magnetic fluid hyperthermia (MFH), cell tracking/loading optimization, nanomagnetic cellular actuation, and nanomagnetic drug and gene delivery, have not yet been reported (For reviews of these applications see [1,4,13]).

Magnetic fluid hyperthermia is a technique that utilizes the coupling of radiofrequency magnetic fields with magnetic nanoparticles to preferentially heat the particles compared to surrounding diamagnetic tissue. As the body is primarily diamagnetic, magnetic fields pass through relatively unimpeded, allowing the applied RF magnetic field to penetrate deep into tissue and deposit significant amounts of energy, primarily but not exclusively in the form of heat, to targets where MNPs have accumulated either via extravasation through leaky tumor vasculature or via biomolecular targeting of cell surface receptors. Herein, we present morphological and magnetic characterization data for Feraheme as well as the results of a preliminary evaluation of the suitability of this SPION for MFH applications.

## 2. Results and Discussions

### 2.1. Transmission Electron Microscopy

**Figure 1:**
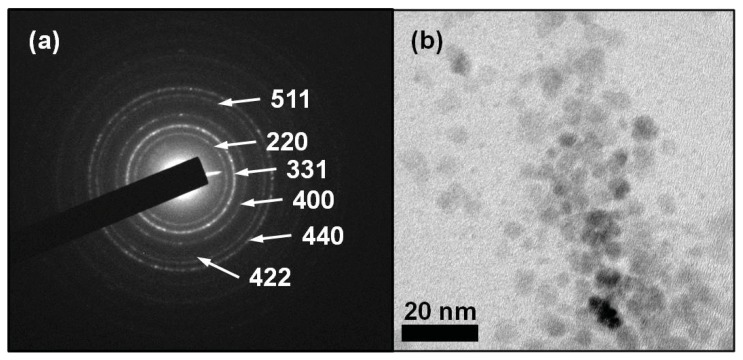
Transmission electron microscopy (TEM) of Feraheme (1:50 dilution with ultra-pure water). (**a**) Selected Area Diffraction image showing a cubic maghemite (γ-Fe_2_O_3_) crystal structure; (**b**) Transmission Electron Micrograph showing electron-dense particle cores < 5 nm in diameter.

**Figure 2:**
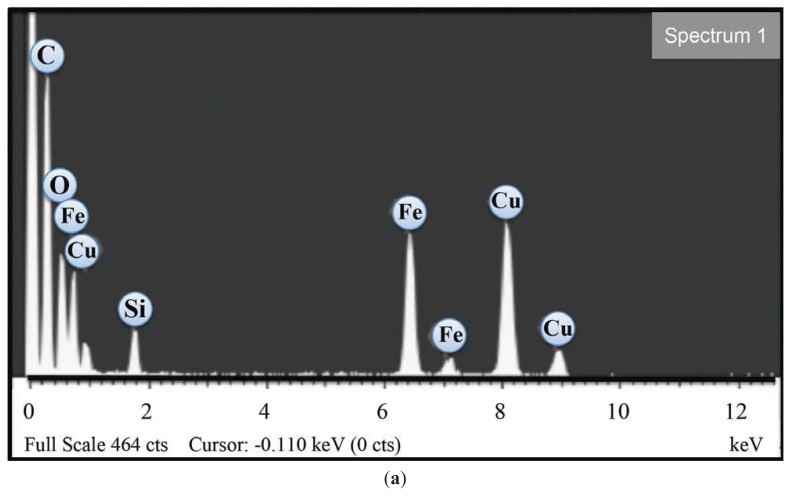

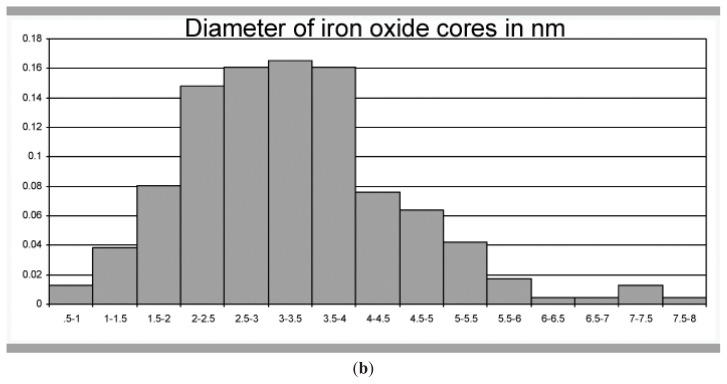
(**a**) Energy Dispersive X-ray elemental analysis of the sample showing iron and oxygen peaks (Cu and Si peaks are from the Formvar-coated copper grids); (**b**) Histogram of iron oxide crystallite particle sizes *vs*. fraction of total (*y*-axis); measured using ImageJ. (**a**)

Selected area electron diffraction analysis is consistent with the lattice spacing of cubic maghemite (γ-Fe_2_O_3_) cores (Figure 1a). The TEM morphological analysis shows that the electron-dense Feraheme cores are irregular in shape (Figure 1b) and have a mean diameter of approximately 3.25 nm. Energy dispersive X-ray analysis confirms the presence of iron and oxygen in an approximately 30:70 atomic ratio (Figure 2a), where the copper and silicon peaks are associated with the Formvar microscope grids and the carbon from the polymer coating. As it is difficult to accurately measure oxygen via EDX due to its low atomic number, this ratio should be considered a rough estimate.

### 2.2. Magnetic Measurements

**Figure 3:**
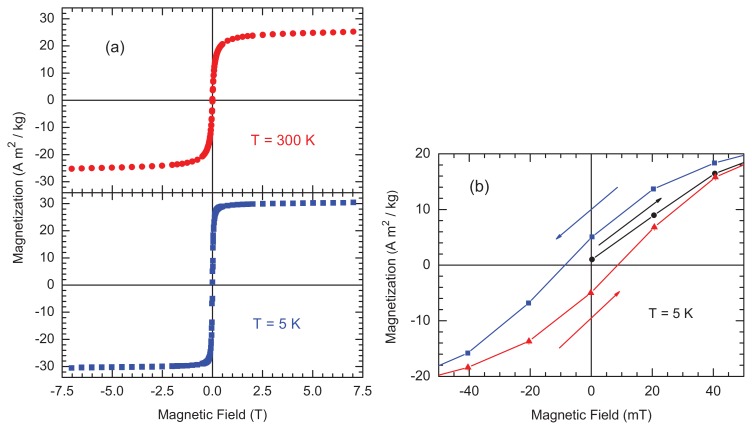
(**a**) Magnetization curves measured at 300 K (top) and 5 K (bottom). (**b**) Low magnetic field region of the hysteresis loop at 5 K. The arrows indicate the directions of the magnetic field sweeps and the lines are guides for the eyes.

Hysteresis loops—a measure of the magnetization in a sample *vs*. applied magnetic field—at 300 K and 5 K show that Feraheme exhibits low coercivity (*i.e*., it is easily magnetized) superparamagnetic behavior at room temperature, consistent with sub-10 nm magnetite/maghemite crystallites as identified by TEM (Figures 1b and 2b). At 5 K, the open loop indicates that at least some of the iron oxide cores are blocked at this temperature, retaining a remanent magnetization even in the absence of an applied field (Figure 3b). The magnetization values observed at 300 K are slightly larger than those reported for ferumoxytol measured in fields up to 2 T [10] and for magnetite nanostructures studied up to 7 T [14].

**Figure 4:**
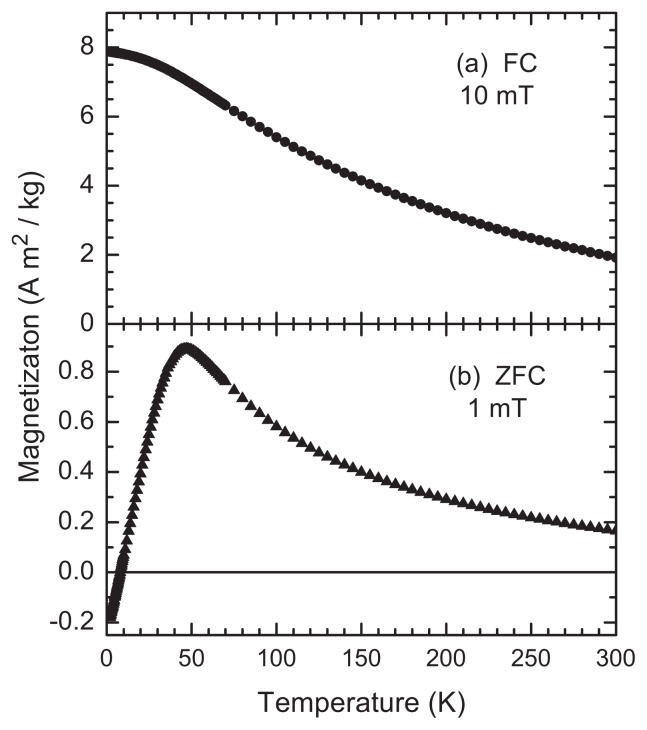
Temperature dependence of the magnetization (**a**) after field cooling (FC) from 300 K in 10 mT and measuring while warming and (**b**) after zero-field cooling (ZFC) from 300 K to 5 K, where a field of 1 mT was applied before measuring while warming.

Though the sample is largely saturated by 250 mT (consistent with the presence of magnetite or maghemite; both low coercivity iron oxides), saturation magnetization—the magnetization at which the electron spins are maximally aligned with the applied field—(*M*_sat_) is not fully achieved at 300 K in 7 T, indicating the possible presence of high-coercivity impurities or oxidation products such as hematite (α-Fe_2_O_3_) and/or goethite (FeOOH). Taking the value at the highest field (*T* = 300 K, B = 7 T) *M*_sat_ = 25.2 Am^2^/kg is just over 1/3 *M*_sat_ for Fe_3_O_4_ [15,16]; however, a direct comparison requires attention to composition, size, and surface properties [17]. The difference between *M*_sat_ for pure maghemite and/or magnetite (*ca*. 80–92 Am^2^/kg) and Feraheme is due to the diamagnetic nature of the polymer matrix within which the iron oxide cores are dispersed. At 300 K, the coercive magnetic field (*H*_c_) could only be bounded to be less than 2 mT. At 5 K, *H*_c_ = 8.5 ± 1.5 mT. The temperature dependence of the magnetization, after field-cooling from 300 K to 2 K while in a continuous field of 10 mT, is shown in Figure 4 and indicates a mean blocking temperature of *ca.* 50 K.

### 2.3. Magnetic Fluid Hyperthermia

Measurement of the heating ability of these particles is important for potential applications in magnetic fluid hyperthermia (MFH)—a technique being investigated for the treatment of solid tumors. MFH relies on the coupling of AC magnetic fields to magnetic nanoparticles (generally iron oxides) dispersed within the tumor. The field applied is of sufficient strength and frequency to induce heating in the particles—primarily via Néel relaxation in superparamagnetic particles [4]. Although the applied field heats the magnetic nanoparticles, the surrounding tissue does not couple to the field in the same way and is left relatively unaffected due to the fact that it is predominantly diamagnetic. This enables the tumor to be specifically targeted even if the field is applied over a relatively large volume of tissue. In practice it has proven difficult to scale up the targeting and distribution of the magnetic nanoparticles within the tumor from small animals to humans. Recent advances in nanoparticle functionalization and cell receptor targeting, however, may provide a way around this drawback [1,4,18].

**Figure 5:**
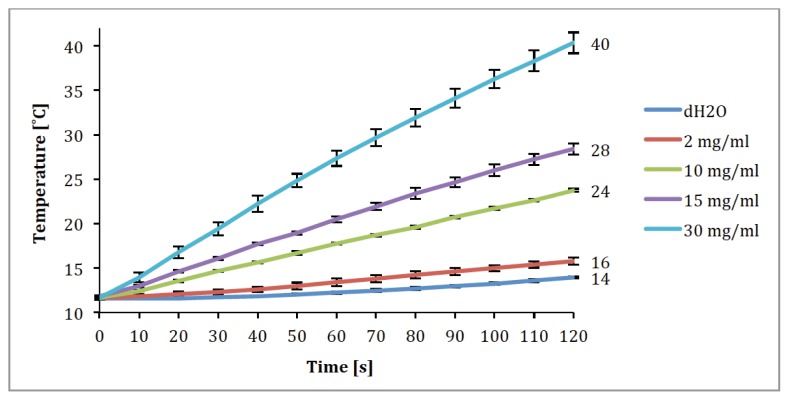
Feraheme suspension temperature rise *vs*. time at concentrations of 2 mg/mL, 10 mg/mL, 15 mg/mL and 30 mg/mL in 1 mL water.

In order to make a preliminary evaluation of this potential, we performed an initial heating experiment in water at 340 kHz and 37.5 kA/m. Results indicate that relatively rapid heating of the Feraheme suspension occurs at varying concentrations between 2 mg/mL and 30 mg/mL. The highest concentration tested (30 mg/mL) produced a temperature increase of 26 °C in 120 s, while the lowest concentration produced a temperature increase of 2 °C over the same time period compared to a water control (Figure 5). These temperature rises equate to a mean Specific Absorption Rates (SAR) of approximately 50.5 W/kg (±17.7 SD). At the lowest concentration, this temperature rise is probably not sufficient to induce cell death. However, at concentrations of 10 mg/mL and higher, the observed temperature rises of more than 10 °C should be sufficient to induce cell death through both apoptosis and necrosis, which occurs with sustained (>30 min) temperature rises of approximately 5 °C above body temperature (For review see [4]).

## 3. Experimental Section

### 3.1. Feraheme Samples

Vials of Feraheme (17 mL) were purchased commercially and consisted of 510 mg of elemental iron per vial (30 mg/mL of iron and 122 mg/mL of particles). Several different lot numbers were used and showed no significant differences in measurements. They were stored at the recommended temperature of 20 °C before use, and were used before the expiration date on the vial. Samples for SQUID magnetometry analysis were freeze-dried. The label states that the material containing the iron oxide particles is “polyglucose sorbitol carboxymethylether”, but no analysis of the organic part was attempted.

### 3.2. High Resolution Transmission Electron Microscopy (HRTEM)

Microscopy was performed on a JEOL 2010F HRTEM at 200 kV. Copper TEM grids were prepared by diluting Feraheme 50 times in double-distilled, deionized water. This solution was then pipetted onto TEM grids, which were placed over filter paper to absorb any excess fluid. The Formvar TEM grids were then covered and allowed to dry overnight. Particle size analysis was conducted by measuring the diameter of 232 particles using NIH ImageJ. Energy dispersive X-ray spectroscopy (EDX) analysis (Oxford Instruments EDS X-ray Microanalysis System coupled to the HRTEM microscope) and selected area electron diffraction were performed on one sample of Feraheme prepared as described above.

### 3.3. Superconducting Quantum Interference Device (SQUID) Magnetometry

Analysis of the magnetic properties of Feraheme was conducted using data acquired with a Quantum Design MPMS XL superconducting quantum interference device (SQUID) magnetometer. A sample of Feraheme was freeze-dried and 11.35 mg (dry weight) was packed into gel caps for insertion into the instrument. The background signal from the gel-caps is known to be significantly less than the signal arising from the sample, so the background was not subtracted. Magnetic hysteresis loops (magnetization [M] *vs*. applied magnetic field [H]) were measured at 5 K and 300 K in fields up to ±7 T. The temperature dependence of magnetization was measured after cooling from 300 K to 5 K in a 10 mT field and subsequently measured while warming in a 10 mT field. Zero-field-cooled magnetization was measured upon warming from 5 K in static field of about 1 mT after cooling from 300 K to 5 K in zero field.

### 3.4. Magnetic Fluid Hyperthermia (MFH)

The suitability of Feraheme for magnetic fluid hyperthermia (MFH) applications was evaluated using an Ambrell AC coil system running at 340 kHz and 37.5 kA/m. Stock Feraheme suspensions were diluted in distilled water to concentrations of 2, 10, 15 and 30 mg/mL (particles) and 1 mL of the new suspensions was then sealed in a sealed PVC vial. The temperature of the suspension was monitored and recorded automatically at 10 s. intervals with an Optical probe (Neoptix Reflex with NEOLink Software) inserted into the suspension. Water without Feraheme particles was evaluated at the same intervals as a control. The starting temperature for both the suspension and the water control was 12 °C. These measurements were repeated four times for each concentration and for the water control.

## 4. Conclusions

Magnetic micro- and nanoparticles are being increasingly employed in biomedical research and, in some cases, clinical diagnostic applications. FDA clearance of Feraheme opens up the possibility of using these particles for biomedical applications other than MRI contrast enhancement. The aim of this study was to better understand the morphological and magnetic properties of these particles in order to begin to evaluate the potential for using Feraheme in clinical applications such as magnetic fluid hyperthermia, *in vivo* cell targeting and actuation, magnetically targeted drug delivery and *in vivo* cell tracking.

The superparamagnetic nature of the magnetic cores described here as well as the biocompatible nature of the polymer matrix makes Feraheme ideal for *in vivo* applications where aggregation of blocked magnetic particles has the potential to cause embolism. The size of the particles allows them to escape the body’s Reticuloendothelial surveillance system (RES), while the relatively high magnetic moment (>25 Am^2^/kg) for a polymer/iron oxide composite, provides an indication that they could be used for intracellular loading in cell targeting applications.

Initial evaluation of the heating ability of Feraheme also indicates that the particles have the potential to be used in magnetic fluid hyperthermia applications. The significant and rapid heating above ambient temperature shown here in the higher concentrations indicate that these particles have the potential to induce cell apoptosis and necrosis if delivered to solid tumors at appropriate concentrations. However, it should be noted that these experiments, like many evaluations of magnetic nanoparticles for hyperthermia applications, were done in water without flow. In the body, blood flow through the tissue provides an effective mechanism for heat transport, likely limiting the clinical effectiveness somewhat or requiring higher MNP concentrations. Another caveat to this analysis is that the starting temperature for these experiments was below body temperature (12 °C). Starting at body temperature would likely result in a slower temperature rise and may not result in the full temperature increase observed over duration of this experiment.

The data provided in this study provide a sound foundation for further evaluation of the potential of Feraheme in biomedical and clinical applications beyond their current use in as MRI contrast agents. The field of Mesoscale Magnetic Biomaterials—magnetic biomaterials that range is size from nanometers to microns—is growing rapidly, with applications spanning from those already in use in the clinic and pathology laboratories, such as MRI contrast and cell separation, to more advanced and futuristic applications in cancer treatment, tissue engineering and regenerative medicine. For all of these *in vivo* applications, the development and use of biocompatible magnetic nanoparticles will be a pre-requisite. In addition, re-purposing formulations that are already in use may provide a shorter route to the clinic for many of these applications. The work presented here represents an initial step in investigating this idea by rigorously characterizing Feraheme and conducting a preliminary investigation for one of these potential future applications—magnetic fluid hyperthermia.
